# Monoclonal Antibodies Against Peptidorhamnomannans of *Scedosporium apiospermum* Enhance the Pathogenicity of the Fungus

**DOI:** 10.1371/journal.pntd.0000853

**Published:** 2010-10-19

**Authors:** Livia C. L. Lopes, Rodrigo Rollin-Pinheiro, Allan J. Guimarães, Vera C. B. Bittencourt, Luis R. Martinez, Wade Koba, Sandra E. Farias, Joshua D. Nosanchuk, Eliana Barreto-Bergter

**Affiliations:** 1 Departments of Medicine and Microbiology and Immunology, Albert Einstein College of Medicine, Bronx, New York, United States of America; 2 Instituto de Microbiologia Professor Paulo de Góes, Universidade Federal do Rio de Janeiro, Rio de Janeiro, Brazil; 3 Gruss Magnetic Resonance Research Center, Albert Einstein College of Medicine, Bronx, New York, United States of America; 4 Departamento de Fisiologia, Universidade Federal do Rio Grande do Sul, Rio Grande do Sul, Brazil; 5 Departamento de Microbiologia e Parasitologia, Universidade Federal do Estado do Rio de Janeiro, Rio de Janeiro, Brazil; Mahidol University, Thailand

## Abstract

*Scedosporium apiospermum* is part of the *Pseudallescheria-Scedosporium* complex. Peptidorhamnomannans (PRMs) are cell wall glycopeptides present in some fungi, and their structures have been characterized in *S. apiospermum*, *S. prolificans* and *Sporothrix schenckii*. Prior work shows that PRMs can interact with host cells and that the glycopeptides are antigenic. In the present study, three monoclonal antibodies (mAbs, IgG1) to *S. apiospermum* derived PRM were generated and their effects on *S. apiospermum* were examined *in vitro* and *in vivo*. The mAbs recognized a carbohydrate epitope on PRM. In culture, addition of the PRM mAbs increased *S. apiospermum* conidia germination and reduced conidial phagocytosis by J774.16 macrophages. In a murine infection model, mice treated with antibodies to PRM died prior to control animals. Thus, PRM is involved in morphogenesis and the binding of this glycopeptide by mAbs enhanced the virulence of the fungus. Further insights into the effects of these glycopeptides on the pathobiology of *S. apiospermum* may lead to new avenues for preventing and treating scedosporiosis.

## Author Summary

The incidence of fungal infections has increased dramatically over the last 50 years, largely because of the increasing size of the population at risk, which especially includes immunocompromised hosts. *Scedosporium apiospermum* is a filamentous fungus that causes a variety of infections, ranging from localized disease to life-threatening disseminated infections. Glycoproteins are molecules present in the fungal surface and are comprised of carbohydrate and protein components. They are involved in different important functions in the fungal cell. Monoclonal antibodies can be used as therapeutic agents for infectious disease, but some factors involved in their efficacy are often not well understood. We found that monoclonal antibodies to glycoproteins present in fungal surface can be nonprotective and can even enhance the disease. The administration of these antibodies can affect functions of the fungal cell and the immune cells, resulting in a survival advantage for the fungus during interactions with the host.

## Introduction

The filamentous and saprophytic fungus *Scedosporium apiospermum* is an emerging clinically important pathogen that causes localized as well as disseminated infections in both immunocompetent and immunocompromised hosts [1]–[2]. *S. apiospermum* is an important cause of mycetoma, acquired by traumatic inoculation. Additionally, the fungus can be acquired through inhalation followed by deposition into the lungs or paranasal sinuses, with similar symptoms to those observed in diseases secondary to *Aspergillus fumigatus*. Dissemination most frequently occurs in immunocompromised individuals and is associated with high mortality rates (>75%) [3]. A large number of cases of pseudallescheriosis/scedosporiosis have been reported in children with cystic fibrosis [4], patients with leukemia [5] and organ transplant recipients [6]–[7]. Despite the rising frequency of *S. apiospermum* infections, its pathogenesis and the mechanism by which *S. apiospermum* evades host pulmonary defenses and reaches other organs are poorly understood. Recently, the innate immune response has been shown to be critical for host defense against *Pseudallescheria* -*Scedosporium* complex fungi [8]. Importantly, these species are largely resistant to traditional antifungals such as amphotericin B; however, newer triazoles, such as voriconazole, can be therapeutic [3].

Microbial adherence is a prerequisite for colonization and an essential step in the establishment of infection [9]. The composition of the fungal cell surface is of primary importance in the cell response to environmental stimuli and, in this context, glycopeptides are important determinants for many biological activities. Elucidation of the primary structure of surface microbial glycopeptides, especially those that function as virulence determinants, is of great relevance to understanding the pathobiology of a microbe. The mechanisms of adherence and invasion have been studied in several fungal species, including *Candida albicans*, *Histoplasma capsulatum*, *A. fumigatus*, *Paracoccidioides brasiliensis*, *Sporothrix schenckii*, *Fonsecaea pedrosoi*, *Trichophyton mentagrophytes* and *Trichophyton rubrum* (reviewed in [9]). However, little is known regarding the adherence and invasion mechanisms for the *S. apiospermum/S. apiospermum* species complex, although their conidia can attached to and are internalized by HEp 2 cells through a lectin-mediated process involving a peptidorhamnomannan of the fungal cell wall [10].

A complex glycopeptide peptidorhamnomannan (PRM) isolated from mycelial forms of *S. apiospermum* has been characterized chemically and immunologically [11]. *S. apiospermum* PRM consists of a peptide chain substituted with both *O*-linked and *N*-linked glycans. It reacts strongly with antiserum against *S. apiospermum* mycelium, and this interaction is weakly inhibited by the PRM from *S. schenckii* or by peptidogalactomannan from *A. fumigatus*, suggesting that *S. apiospermum* expresses antigens that are related to *S. schenckii* peptidopolysaccharide [12] and the major *Aspergillus* glycopeptide [11], [13].

To gain a better understanding of PRM function in *S. apiospermum*, we generated murine monoclonal antibodies (mAbs) against PRM. Interestingly, the mAbs promoted conidial germination. Infection of macrophage monolayers with opsonized *S. apiospermum* conidia resulted in a significant increase in the killing of macrophages and a decrease in phagocytosis in comparison with non-opsonized conidia. Mice that received the mAbs prior to *S. apiospermum* infection died more rapidly than control animals. These results suggest that mAbs to PRM change the physiology of *S. apiospermum* cells by altering the kinetics of germination and modifying fungal-host interactions, which dramatically impacts the outcome of disease.

## Materials and Methods

### Microorganism and growth conditions

*S. apiospermum* strain HLPB (formerly *Pseudallescheria boydii*), isolated from a patient with eumycotic mycetoma, was kindly supplied by Dr. Bodo Wanke from Instituto de Pesquisa Evandro Chagas, Fundação Oswaldo Cruz, Rio de Janeiro, Brazil. The isolate was confirmed as *S. apiospermum* by molecular methods developed by Dr. Kathrin Tintelnot (Robert Koch-Institut, Berlin, Germany). The sequencing of the ITS regions revealed that this strain belongs to clade 4 (*S. apiospermum* sensu stricto) according to the taxonomy proposed by Gilgado *et al.* [14]. Cells were maintained on potato dextrose (PD) agar slants. Fresh cultures were inoculated in PD liquid culture medium and incubated for 7 days at 25°C with shaking. Conidia were grown on Petri dishes containing PD agar medium at 30°C. After 7 days in culture, conidia were obtained after washing the plate surface with phosphate-buffered saline (PBS- 10 mM NaH_2_PO_4_, 10 mM Na_2_HPO_4_ pH 7.0 and 150 mM NaCl) and filtering through gauze to remove hyphal fragments and debris. Conidia were washed three times and counted with a hematocytometer.

*S. apiospermum* clade 5 (*S. apiospermum* sensu stricto) [14] and *Scedosporium prolificans* strains were kindly supplied by Dr. J. Guarro from Unitat de Microbiologia, Facultat de Medicina e Institut d'Estudis Avançats, Réus, Spain. Cells were maintained on Sabouraud (SAB - 2% glucose, 1% peptone, 0.5% yeast extract) agar slants. Fresh cultures were inoculated in SAB liquid culture medium and incubated for 7 days at 25°C with orbital shaking. Conidia were grown on Petri dishes containing SAB agar medium at 30°C. After 7 days in culture, conidia were obtained as described for *S. apiospermum*. *Candida albicans* SC5314 (ATCC, MYA-2876), *Candida parapsilosis* GA1 [15], and *Histoplasma capsulatum* G217B (ATCC, 26032) were maintained at −80°C in 35% glycerol. Yeast phases of the *Candida* species were produced in YPD (1% yeast extract, 2% bactopeptone, 2% glucose) at 30°C and *H. capsulatum* yeasts were grown in YPD at 37°C.

### Reagents and cell lines

A goat anti-mouse (GAM) IgG (Southern Biotechnology Associates Inc., Birmingham, AL) was used as an isotype-matched control in all the experiments. The fluorescence probe 5-(and 6)-carboxytetramethylrhodamine succinimidyl (NHSRho) was obtained from Molecular Probes (Eugene, OR). Triton X-100, fluorescein isothiocyanate (FITC)-dextran (molecular weight, 70,000), MTT [3-(4,5-dimethyl-thiazol-2-yl) 2,5-diphenyl tetrazolium bromide], and paraformaldehyde were from Sigma-Aldrich (St. Louis, MO). Tetramethyl Rhodamine Isothiocyanate (TRITC) was obtained from Southern Biotechnology Associates Inc. SuperBlock buffer in phosphate-buffered saline (PBS) and EZ-Link sulfo-N-hydroxysulfosuccinimide – biotin kit were from Pierce (Rockford, IL). The macrophage-like cell line J774.16 (derived from a reticulum cell sarcoma) was obtained from the ATCC. The J774.16 cells were grown with DMEM (Life Technologies, Carlsbad, CA) containing 10% fetal calf serum (Gemini Bio-Products, Woodland, CA), 10% NCTC-109 (Life Technologies), 1% nonessential amino acids (Mediatech, Manassas, VA) and 1% Penicillin-Streptomycin (Invitrogen, Carlsbad, CA) at 37°C in 5% CO_2_. The cell counts and viability for all the experiments were determined by trypan blue vital dye exclusion using a hemacytometer. For conidia, this method demonstrated an initial viability of >95%, as confirmed by plating. PRM was produced as described [11].

### Generation of mAbs against *S. apiospermum* peptidorhamnomannans

All murine studies were performed in accordance with the rules and regulations of animal welfare at Federal University of Rio de Janeiro (UFRJ, RJ, Brazil) and the Albert Einstein College of Medicine (Bronx, NY, USA). Four 6 week old female BALB/c mice (from UFRGS,RS, Brazil) were immunized intraperitoneally with 20 µg of PRM emulsified in complete Freund's adjuvant for the first injection and 20 µg of antigen in incomplete Freund's adjuvant for the subsequent three injections. Injections were spaced at a two-week intervals and the immune response against PRM was monitored by indirect ELISA. Sera were obtained 1 week after the last immunization and analyzed for the presence of antibodies to PRM by ELISA, using 500ng of the antigen per well. The animal whose serum showed the highest level of immunization (OD value 6.5 times higher than the serum of a non-immunized animal) was boosted intraperitoneally with 50 µg of the antigen without adjuvant 3 days prior to spleen removal and fusion of splenocytes with murine myeloma tumor cells (SP2/0) using polyethylene glycol (PEG). Hybridomas that survived selection in hypoxanthine-aminopterin-thymidine (HAT) medium were screened for antibody production by ELISA using 100 ng of the antigen per well. Positive hybridomas were cloned by limiting dilution and cryopreserved. The isotype of the selected mAbs was determined with an isotyping kit (Sigma-Aldrich) according to the manufacturer's instructions. The murine mAbs C7, C11 and F10 of immunoglobulin G1 (IgG1) isotype were selected and used in all assays. Clones were injected into the peritoneal cavity of BALB/c mice previously treated with Pristane to generate ascites and the antibodies to PRM were subsequently purified by protein G affinity chromatography. The purified mAbs were screened to ensure the absence of endotoxin with a *Limulus* amebocyte assay kit (BioWhittaker Inc., Walkersville, MD).

### Indirect ELISA

PRM was added (25–50 ng of protein in 50 µL of PBS [0.01M; pH 7.2]) per well or 1×10^6^ swollen conidia (*S. apiospermum* strain HLPB (clade 4), *S. apiospermum* strain clade *5* and *S. prolificans*) or yeasts (*C. albicans*, *C. parapsilosis* and *H. capsulatum*) in 50 µl PBS per well, followed by incubation for 1h at 37°C and then overnight at 4°C. Plates were washed three times with washing buffer (10 mM Tris-buffered saline [TBS], 0.1% Tween 20 [pH 7.3]) and blocked with 1% BSA in PBS (blocking buffer). Serial two-fold dilutions of a 100 µg/mL solution of the different mAbs in blocking buffer were added in duplicate to the wells and incubated at 37°C for 1 h. After three washes, the plates were incubated at 37°C for 1 h with GAM IgG alkaline phosphatase conjugate (Southern Biotech, Birmingham, AL) diluted 1∶1,000 in blocking buffer at a final volume of 100 µL per well. Plates were washed three times, and then the enzymatic reaction was developed with the addition of pNPP in substrate buffer at 37°C for 30 min. Absorbances were measured on a microplate reader (Bio-Tek μQuant) at 405 nm.

### Fluorescence microscopy

Immunofluorescence analysis was performed by co-incubating the mAbs with *S. apiospermum* conidia. In order to assess mAb binding, conidia were incubated in SuperBlock for 1 h at 37°C, washed three times with PBS and incubated with either a mAb to PRM or an isotype-matched control in 100µg/mL in SuperBlock for 1 h at 37°C. The cells were washed and incubated in 100 µl of GAM IgG conjugated with TRITC at a 1∶100 dilution in SuperBlock for 1 h at 37°C. After three washes, cells were suspended in 50 µL of a mounting solution containing 0.01 M of *N*-propylgallate diluted in PBS∶glycerol (1∶1, vol/vol). Ten microliters of the suspension was applied to a microscope slide and examined with an Olympus AX70 fluorescence microscope (Olympus America Inc., Center Valley, PA) using a 620-nm filter and a magnification of ×40.

### Competition ELISA

MAbs were biotinylated with a biotin commercial kit, according to the manufacturer's instructions (Pierce, Rockford, IL, USA). ELISA plates were generated as described above, except the concentration of PRM was 25ng/well. After blocking, a constant concentration of the biotinylated mAb was incubated with decreasing concentrations of a different non-biotinylated mAb in blocking buffer for 1 h at 37°C. After washing, avidin conjugated with alkaline phosphatase (Sigma-Aldrich) was added, and the preparation was incubated for 1 h at 37°C. Absorbance at 405 nm was recorded after the reaction was developed with *p*NPP.

### Epitope characterization of mAbs by endoglycosidase treatment

To remove N-linked glycans, 10 µg of PRM was treated with 100mU of PNGase F (P0704, New England Biolabs) at 37°C for 20 h. ELISA plates were made with 25 ng/well of the treated PRM, the mAbs were applied, and the reaction developed as described above.

### Epitope characterization of mAbs by protease digestion

ELISA plates were made with 25ng/well of purified PRM and the wells were incubated with Proteinase K (2.5 µg/mL; Sigma-Aldrich) in 1% SDS solution for 1 h at 4°C [16]. After washing, the mAbs were applied and the reaction developed as described above.

### Phagocytosis assays

Phagocytosis assays were performed as described previously [17]. Briefly, macrophage-like J774.16 cells were plated at a concentration of 5×10^5^ cells per well in 24-well cell culture polystyrene plates and grown overnight at 37°C in the presence of 5% CO_2_. *S. apiospermum* conidia were collected after 7 days of growth, washed three times with PBS and 1×10^6^ conidia were incubated with 100 µg/mL of a mAb to PRM, IgG isotype control, or PBS for 1 h at 37°C. After washing, the fungal cells were added to the macrophages at a ratio of 5∶1 (conidia∶macrophage), and the plates were incubated for 1 h at 37°C in the presence of 5% CO_2_. Samples were prepared in triplicate. Wells were washed with PBS and fixed with a 40% methanol solution. The numbers of macrophages and conidia were recorded for each field, and at least 200 macrophages were counted. The phagocytosis index was defined as the ratio of the number of intracellular conidia relative to the number of macrophages counted. A second phagocytosis experiment was performed with J774.16 cells that were incubated with native PRM (100 µg/mL) 1 h before the interaction with *S. apiospermum* conidia.

To determine the mechanism of conidial engagement with macrophages, additional phagocytosis experiments were performed using fluorescence activated cell sorting (FACS). J774.16 cells were incubated with anti-mouse CD11a, anti-mouse CD11b, anti-mouse CD11c, anti-mouse CD14 or anti-mouse CD18 (Southern Biotechnology Associates Inc.) antibodies prior to interaction with *S. apiospermum* conidia. The conidia were incubated in a solution of 0.5 mg/mL of FITC in PBS at 37°C for 30 min The conidia were then washed three times with PBS and incubated with treated and control macrophages for 60 min. Samples were washed three times with PBS to remove extracellular conidia. For FACS measurements, cells were suspended in 1 mL PBS and analyzed on a FACS-Calibur™, equipped with a 5 W argon laser (Coherent) tuned to 488 nm, output power 250 mW (Becton Dickinson, San Jose, CA). At least 10,000 events were enumerated for each condition [18].

### Macrophage effector functions

The growth of *S. apiospermum* conidia in the presence of mAbs was evaluated by incubating the fungus with mAbs to PRM, isotype-matched control mAb, or PBS prior to co-culture with macrophages. Washed conidia were added to wells containing J774.16 cells at a ratio of 5∶1 and incubated for 2 h. The cultures were washed with cold PBS, and the macrophages were lysed by adding sterile water. Aliquots were plated into potato dextrose agar plates and incubated at 30°C. The percentage of growth was determined by comparing the number of CFU for *S. apiospermum* conidia pretreated with mAbs to the number of CFU for untreated conidia.

### Assay for phagolysosome formation

To further explore whether the mAbs affected the intracellular fate of the fungus in macrophages, fusion of phagosomes and lysosomes was evaluated as described [19]. Monolayers of J774.16 cells were incubated in fresh non-phenol-red medium with 0.5 mg/mL FITC-dextran for 4 h at 37°C in the presence of 5% CO_2_. Cells were washed three times with PBS and incubated overnight in medium alone. *S. apiospermum* conidia were collected, washed, and incubated with 40 µg/mL NHSRho at 4°C for 1 h. Conidia were washed and incubated with 100 µg/mL of mAb to PRM, control mAb or PBS. Conidia were washed, suspended in DMEM, and added to the culture of J774.16 cells at a ratio of 5∶1. The plates were then incubated for 1 h at 37°C in the presence of 5% CO_2_. The cells were fixed in 3.75% paraformaldehyde for 20 min at room temperature. Cells were observed by phase-contrast and fluorescence microscopy at a magnification of ×400. In J774.16 macrophages, the number of rhodamine-labeled *S. apiospermum* conidia with co-localization of FITC-dextran and the total number of intracellular labeled conidia for each condition were counted to determine the percentage of phagosomes fused with lysosomes, which were characterized by red fluorescently labeled conidia co-localized with a green fluorescent dextran ring. To evaluate the capacity of *S. apiospermum* conidia to germinate and survive in acidic media, cells were incubated for 4 h in DMEM at pH 7.2 or pH 4.0, and the number of germinated conidia was determined.

### Time-lapse microscopy

For live cell imaging, phagocytosis was carried out as described above. Briefly, 5×10^4^ macrophages were plated on polylysine coated coverslip bottom MatTek plates and allowed to adhere overnight. The media was then removed and replaced with fresh media containing *S. apiospermum* conidia (*S. apiospermum* to macrophage ratio of 5∶1). This assay was also performed with conidia opsonized with mAbs F10, C7, C11 and irrelevant mAb (100 µg/mL). Macrophages and conidia were incubated together for 1 h to allow for completion of phagocytosis, washed once with fresh media, replenished with 2 mL feeding media and followed by time-lapse imaging every 10 min. Images were collected at 10× using the Axiovert 200 M inverted microscope and photographed with an AxiocamMR camera controlled by the Axio Vision 4.4 software (Carl Zeiss Micro Imaging, NY). This microscope was housed in a Plexiglas box and the temperature was stabilized at 37°C with a forced air heater system. The plate lid was kept in place to prevent evaporation, and 5% CO_2_ was delivered to a chamber locally at the culture dish. Movie animations were created using ImageJ software [20].

### Germination assay

The germination assay was performed as described with minor modifications [21]. *S. apiospermum* conidia (1×10^5^/mL) were incubated in DMEM in 24-wells plates at 37°C with 100 µg/mL of mAb to PRM, control mAb or PBS. At 2, 3, 4, 8 and 24 h the wells were analyzed and germinated conidia were counted by microscopy. At least 100 conidia per field were counted, and the mean value of three independent counts was calculated. Percent germination was calculated as germinated conidia/total counted conidia ×100.

### Measurement of nitric oxide and superoxide release by macrophages

J774.16 cells were plated at 10^5^ cells per well in 96-well polystyrene tissue-culture plates. Conidia were pre-incubated with mAb to PRM, nonspecific IgG, or PBS for 1 h at 37°C prior to addition to the macrophage monolayer. After 1 h of incubation, aliquots from the supernatant were collected at different intervals. Nitric oxide levels were measured using a commercial Griess reagent kit (Promega). Similarly, superoxide dismutase activity was determined using a method that involves generation of superoxide and reduction of the tetrazolium dye MTT to its formazan, which is measured at 570 nm [22].

### Survival studies

For survival studies, groups of 6 BALB/c mice (National Cancer Institute (NCI), Frederick, MD) were injected intraperitoneally with either 250 µg of a mAb to PRM, an isotype-matched control mAb, or PBS. MAb F10 was also used at 100 and 500 µg per mouse. Two h later, the mice were intratracheally infected with 2.5×10^7^ *S. apiospermum* conidia. A similar model was tested using intravenous inoculation with 1.25×10^6^ *S. apiospermum* conidia. Mice were monitored closely and their survival determined.

For survival studies using a model of invasive candidiasis, 6- to 8-week-old A/J (NCI) mice were inoculated intraperitoneally with 250 µg of mAb F10, an IgG isotype-matched control, or PBS 2 h prior to intravenous injection of 1×10^6^ *C. albicans* yeast cells. Animals were euthanized at day 7 after infection and the kidneys were removed, weighed, homogenized and plated onto YPD agar at 30°C for CFU determinations.

### Heterologous yeasts (*Candida albicans*) phagocytosis with mAb to *S. apiospermum* PRM

Phagocytosis assays were performed as above, and *C. albicans* yeasts were added to macrophage monolayers (5∶1 yeasts∶macrophage). Additionally, a second phagocytosis experiment was performed with J774.16 cells that were incubated with native PRM (100 µg/mL) 1 h before the addition of yeast cells.

### Statistical analysis

Statistical analyses were performed using GraphPad Prism version 5.00 for Windows (GraphPad Software, San Diego CA). Unless otherwise noted, a one-way analysis of variance using a Kruskall-Wallis nonparametrical test was used to compare the differences between groups, and individual comparisons of groups were done using a Bonferoni posttest. A *t* test was used to compare the number of CFU for different groups. A 90–95% confidence interval was determined in all experiments. Survival results were analyzed by a Kaplan-Meyer test to determine the differences between groups.

## Results

### Binding of mAbs to *S. apiospermum* PRM

**Figure 1:**
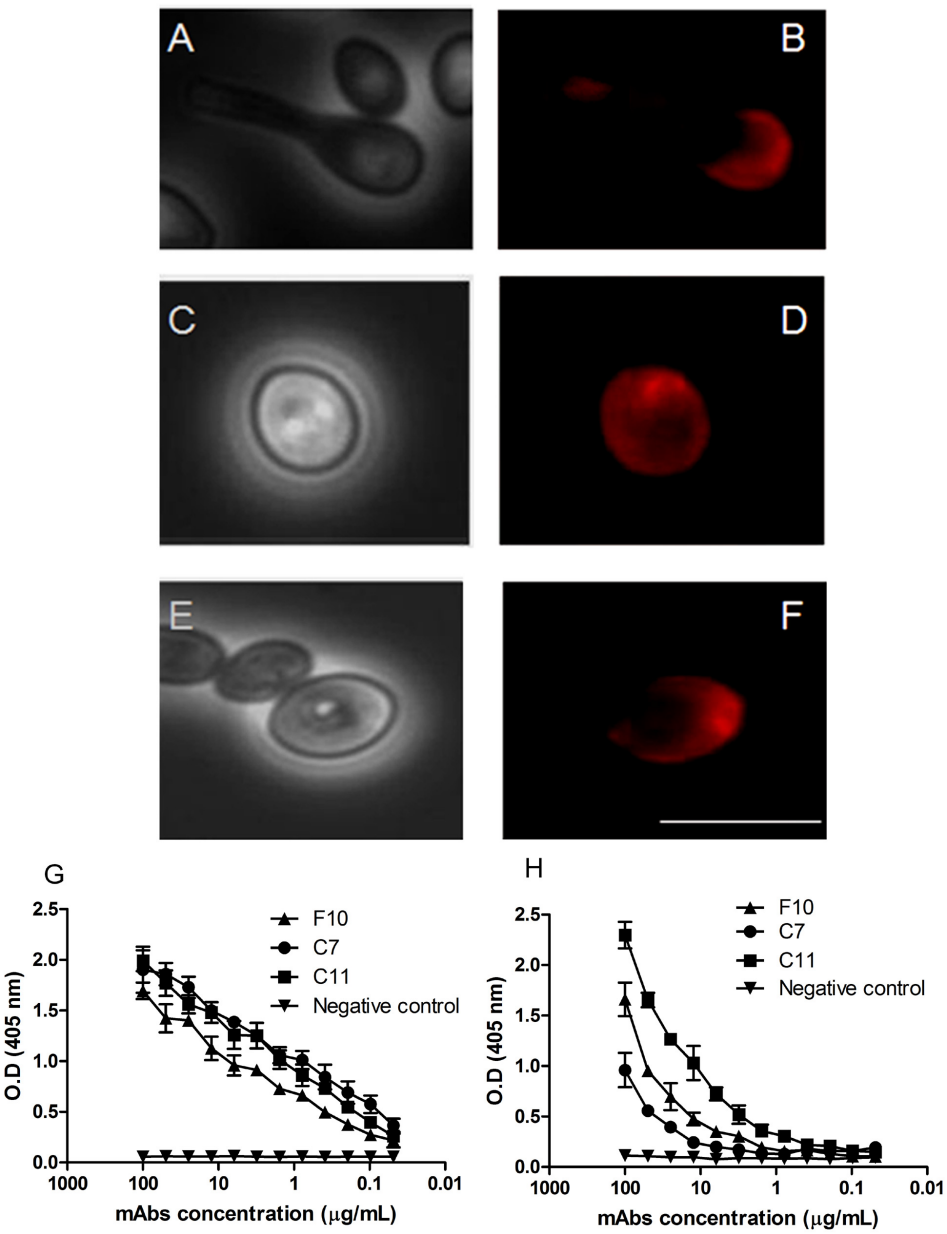
MAb-labeled PRM on the cell surface of *S. apiospermum*. Immunofluorescence and bright-field microscopy showing labeling of *S. apiospermum* by mAbs to PRM. Representative images of binding with mAbs F10 (A and B), C7 (C and D) and C11 (E and F). Bars: 10µm. Representative curves for mAb binding to PRM of purified PRM (G) and of *S. apiospermum* conidia (H) as determined by indirect ELISA. The ELISA assays were done in triplicate, three times.

Three IgG1 mAbs, C7, C11, and F10, were generated from a mouse immunized with PRM. Immunofluorescence microscopy revealed that the three mAbs against PRM could bind resting conidia, swollen conidia and hyphae, but only mAb F10 bound germinating conidia, labeling the cell body and the apical portion of germinative tube (Figure 1A and B). Fluorescence images of mAbs C7 (Figure 1C and D) and C11 (Figure 1E and F) show labeling of swollen conidia. An indirect ELISA was used to quantitatively evaluate mAb binding to PRM. There were variations in binding by the different mAbs and the relative order of reactivity was C7≥C11>F10 (Figure 1G). These mAbs can recognize native PRM (Figure 1G) and fixed swollen conidia by ELISA (Figure 1H), suggesting that the mAbs recognize epitopes exposed on the native structure of PRM on the conidia cell surface.

**Figure 2:**
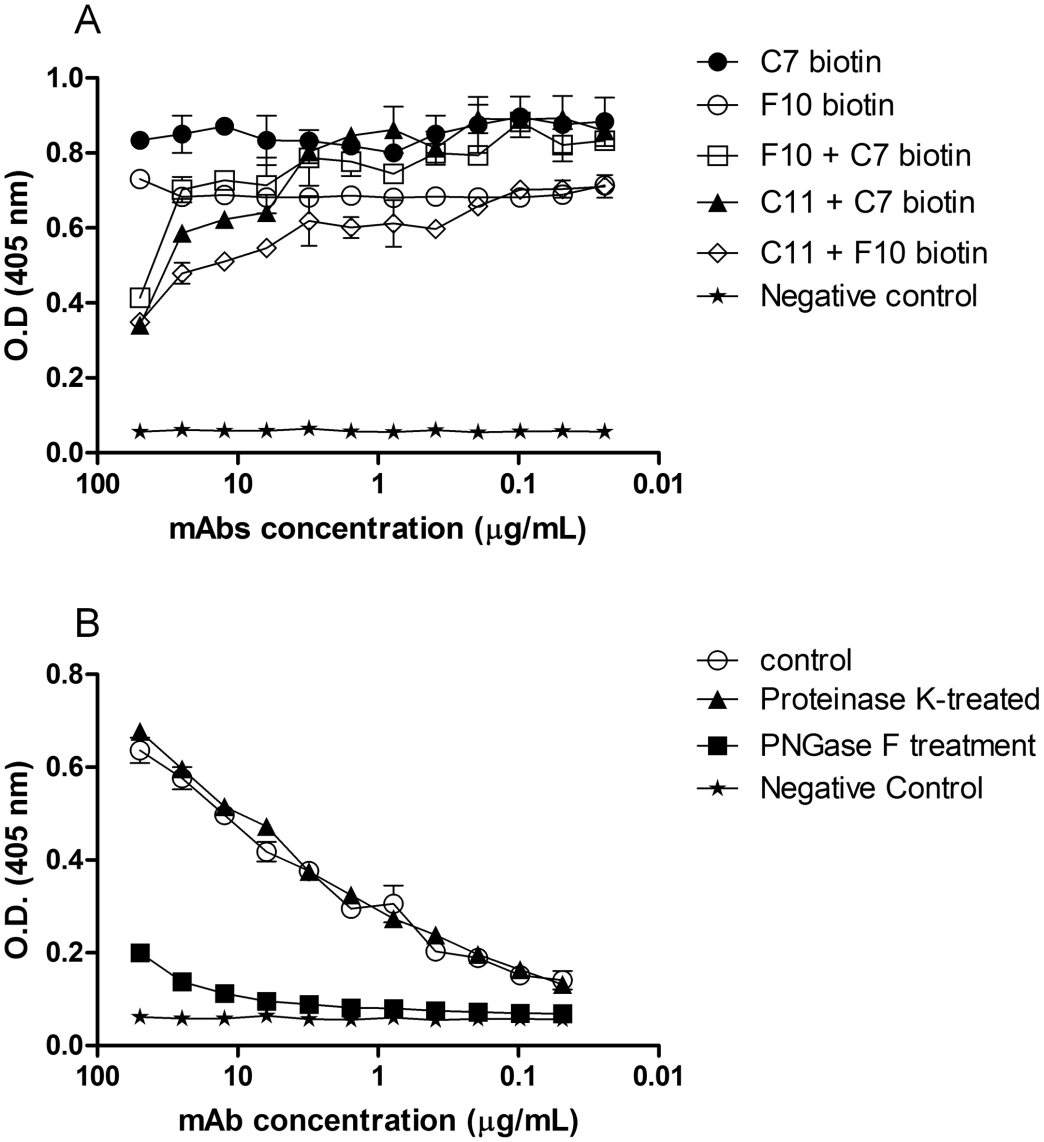
MAbs to PRM compete for the same epitope in the carbohydrate portion of PRM molecule. Competition ELISA showing the competition between mAbs F10, C7 and C11 (A). Absorbance values from ELISA with endoglycosidase-treated antigens and absorbance values from ELISA with protease-treated antigens incubated with mAb F10 (B). The ELISA assays were done in quintuplicate, three times.

Competition ELISA assays showed that the three mAbs to PRM bind extremely close or overlapping epitopes (Figure 2A). Indirect ELISAs using native PRM treated with PNGase F or proteinase K in 1% SDS solution were used in order to analyze which portion of the glycoprotein is recognized by the mAbs. Removal of the carbohydrate from PRM significantly reduced the binding of mAb F10 to PRM (p<0.05) (Figure 2B). In contrast, no significant difference was observed when the protein portion was removed, showing that the epitopes do not contain protein moieties. Similar results were observed for mAbs C7 and C11 (data not shown).

### Binding of mAbs to PRM with other fungi

Indirect ELISAs were performed using different fungal cells to evaluate the specificity of the mAbs. Interestingly, the mAbs to PRM recognized conidial forms of *S. apiospermum* clade 5 (Figure S1A) and *S. prolificans* (Figure S1B) as well as yeast forms of *H. capsulatum* (Figure S1C), *C. albicans* (Figure S1D) and *C. parapsilosis* (Figure S1E). Hence, there appears to be conserved mannose-containing structures on the cell surfaces of these fungi.

### MAbs to *S. apiospermum* PRM alter effector functions of macrophages *in vitro*

**Figure 3:**
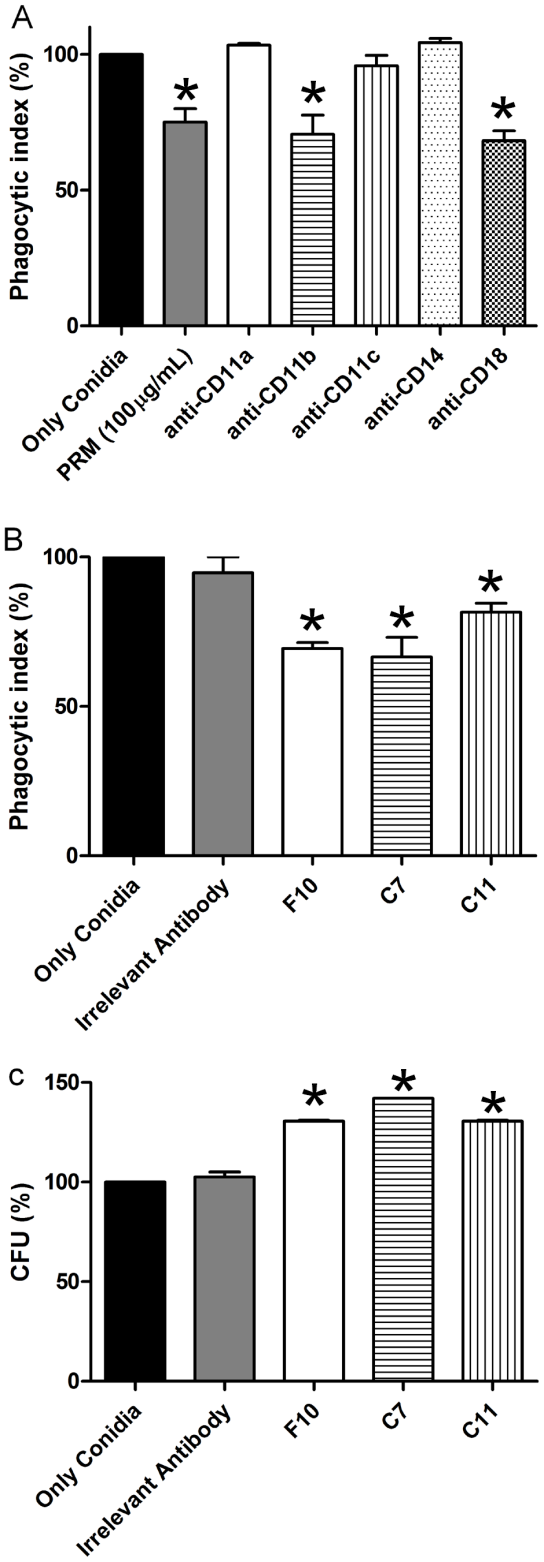
MAbs modify the intracellular fate of *S. apiospermum*. Effect of purified PRM and blockage of the CD11 and CD18 receptors decreased phagocytosis of *S. apiospermum* conidia (A) and mAbs to PRM (B) on *S. apiospermum* phagocytosis by J774.16 cells. Effect of mAbs to PRM on *S. apiospermum* killing by J774.16 cells (C). In all the panels, the values are the averages of three independent experiments, and the error bars indicate standard deviations. Each experiment was done in triplicate. * *P*<0.05 for comparison between samples and controls.

*S. apiospermum* conidia are effectively phagocytosed by J774.16 macrophages. The addition of soluble PRM significantly reduced phagocytosis by 25% (Figure 3A). To investigate the involvement of cellular receptors on conidia uptake, specific blockers were used. A significant reduction in the phagocytosis of *S. apiospermum* conidia was observed using either anti-CD11b or anti-CD18 antibodies, indicating a requirement for CR3 in the interaction between conidia and macrophages (Figure 3A). Furthermore, mAbs F10, C7, and C11 significantly decreased the phagocytosis of conidia compared to conidia incubated with PBS or an isotype-matched IgG control mAb by 31, 33 and 19%, respectively (*P*<0.05) (Figure 3B), suggesting blocking of phagocytosis by these mAbs. Although phagocytosis was reduced by the mAbs, there were significant increases of 30–40% in the intracellular survival of phagocytosed *S. apiospermum* when compared with PBS or an isotype-matched control mAb (Figure 3C).

### Phagosomal maturation and lysosomal fusion

**Figure 4:**
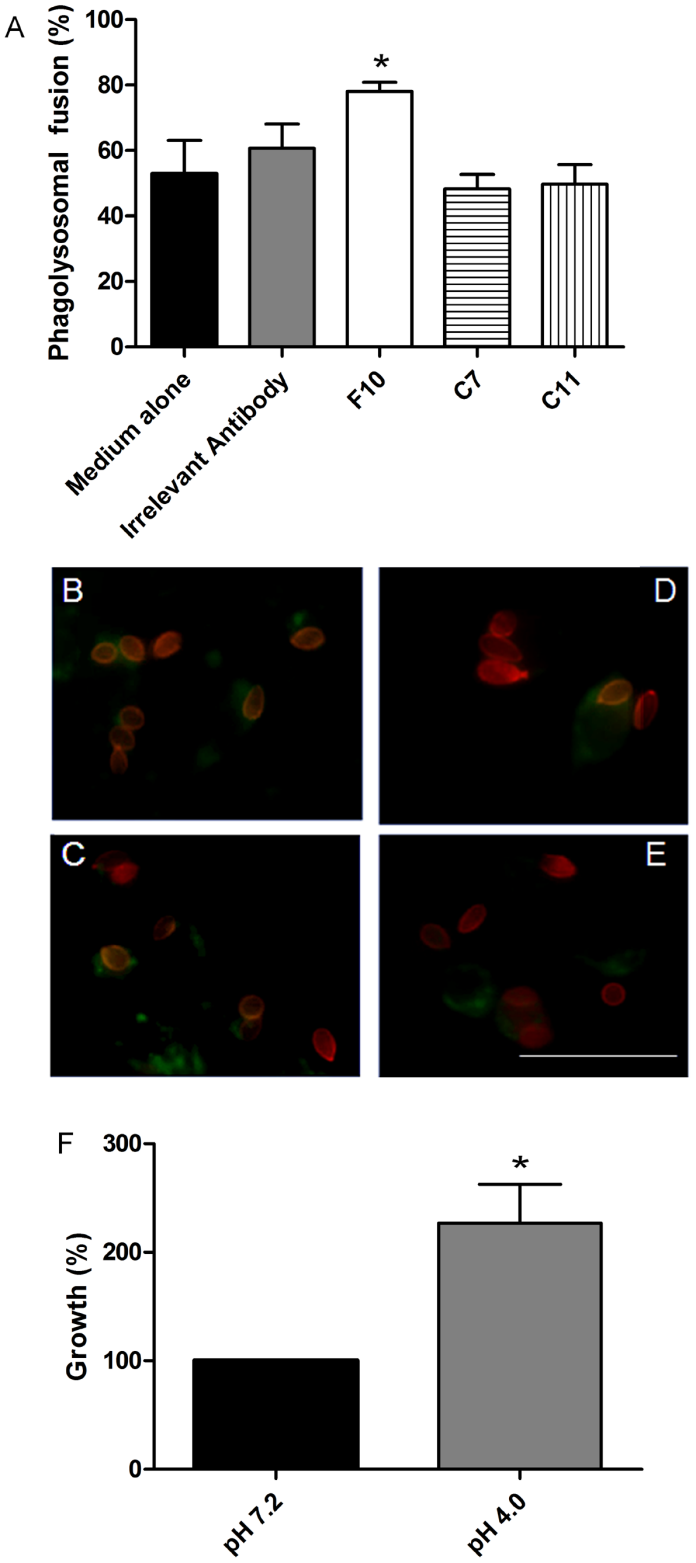
Phagolysosomal fusion with *S. apiospermum*. Localization of *S. apiospermum* conidia in phagolysosomes. MAb F10 increased the phagolysosomal fusion in J774.16 cells (A). Immunofluorescence analysis of phagolysosomal fusion in J774.16 macrophages by FITC-dextran colocalization with NHS-Rho-labeled *S. apiospermum* conidia. *S. apiospermum* conidia (red) previously incubated with mAb F10 (B), C7 (C), C11 (D) and irrelevant control mAb (E) with a J774.16 cell with FITC-dextran (green). Growth of *S. apiospermum* conidia in neutral and acidic medium (DMEM) for four h (F) (* *P*<0.05). Both experiments were performed three times. Scale bar: 50 µm.

Fusion of lysosomes with phagosomes was observed by the detection of FITC-dextran with *S. apiospermum conidia* within macrophages. The majority of phagosomes containing *S. apiospermum* opsonized with mAb F10 demonstrated co-localization of FITC-dextran with NHSRho-labeled conidia (Figure 4A and B), which was significantly increased compared to the other conditions examined, including mAb C7 (Figure 4A and C), mAb C11 (Figure 4A and D) and irrelevant control mAb (Figure 4A and E). In order to evaluate the capacity of *S. apiospermum* conidia to germinate and survive in acidic media, cells were incubated in DMEM at pH 7.2 or pH 4.0. Interestingly, there was a significant increase (127%) in the number of germinated conidia at pH 4.0,compared to pH 7.2 (Figure 4F).

### Interaction between *S. apiospermum* conidia and J774.16 cells

In order to evaluate if macrophages were able to eliminate the fungus, *S. apiospermum* conidia was co-cultured with J774.16 cells. We observed that the macrophages could not eradicate the *S. apiospermum* (Video S1). Further, *S. apiospermum* conidia could germinate inside and destroy the macrophages (Video S1). The same assay was performed with conidia opsonized with mAbs F10, C7 and C11, and similar results were achieved (data not show).

### Metabolic activity and germination of *S. apiospermum* conidia in the presence of mAbs to PRM

**Figure 5:**
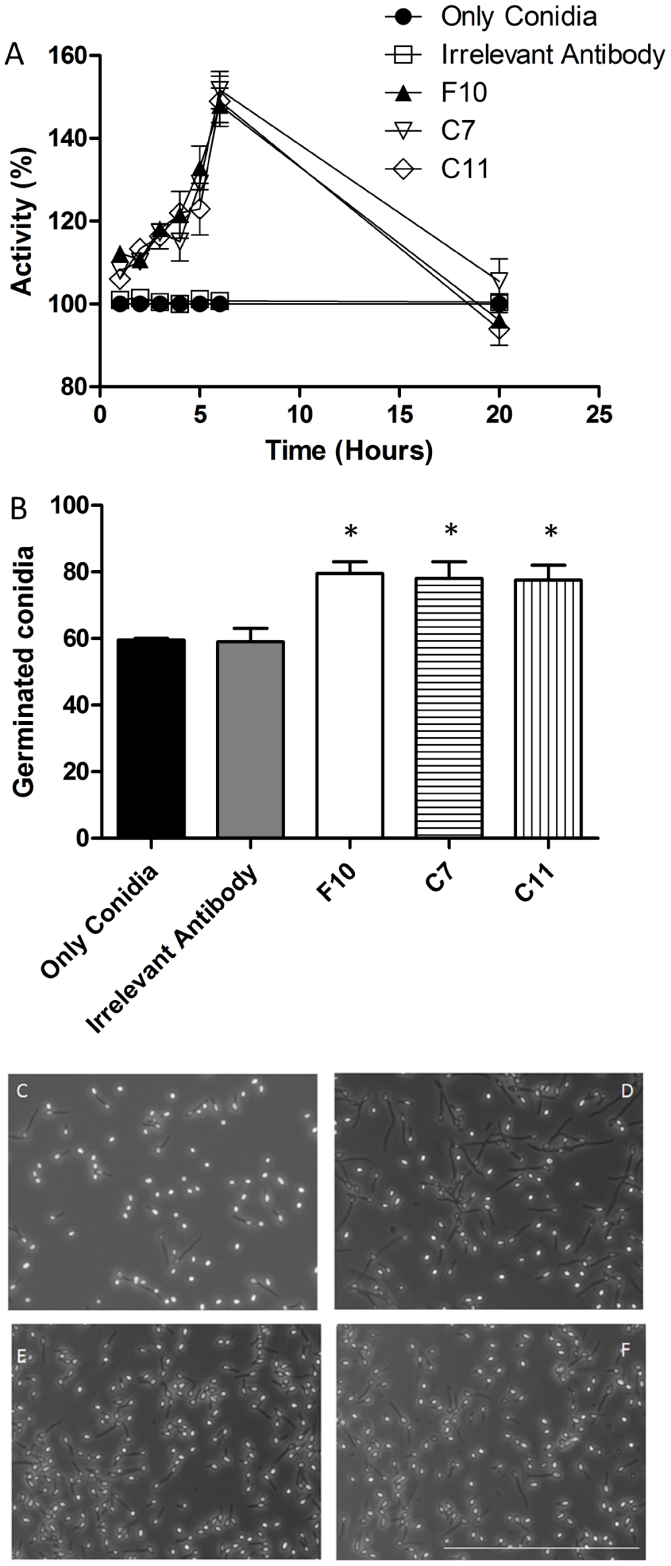
Effect of mAbs to PRM on the growth of *S. apiospermum* conidia. Viability of *S. apiospermum* in the presence of mAbs against PRM was accessed by MTT assay (A). Effect of mAbs against PRM on *S. apiospermum* morphogenesis (B–F). Percentage of cells producing germ tubes after 4 h (B). Morphology of *S. apiospermum* cells assessed by light microscopy after incubation with an irrelevant antibody (C) and with mAbs F10 (D), C7 (E), C11 (F). There is a stimulus in the germination, when mAbs are present (B–F). Bars in the panels represent standard errors and * *P*<0.05. Scale bar: 100µm. All the experiments were performed three times.

The metabolic activity in *S. apiospermum* conidia in the presence of the three mAbs against PRM was examined by MTT reduction assay at 1, 2, 3, 4, 5, 6 and 20 h (Figure 5A). The greatest increase in activity was at 6 h, where the increase was 48%, 49% and 51% for F10, C7 and C11, respectively, in comparison with controls. There were no differences at 20 h (p>0.05), which is consistent with the germination of all viable conidia in medium alone by this time.

The influence of mAbs F10, C7 and C11 mAbs on conidia germination *in vitro* was examined. The evaluation was based on visible germinative tube formation. After 4 h of incubation, 77–80% germination was observed in the presence of each of the mAbs to PRM, compared to 59% germination for controls (Figure 5B and 5C–F).

### Nitric oxide and superoxide release by J774.16 macrophages-like cells

**Figure 6:**
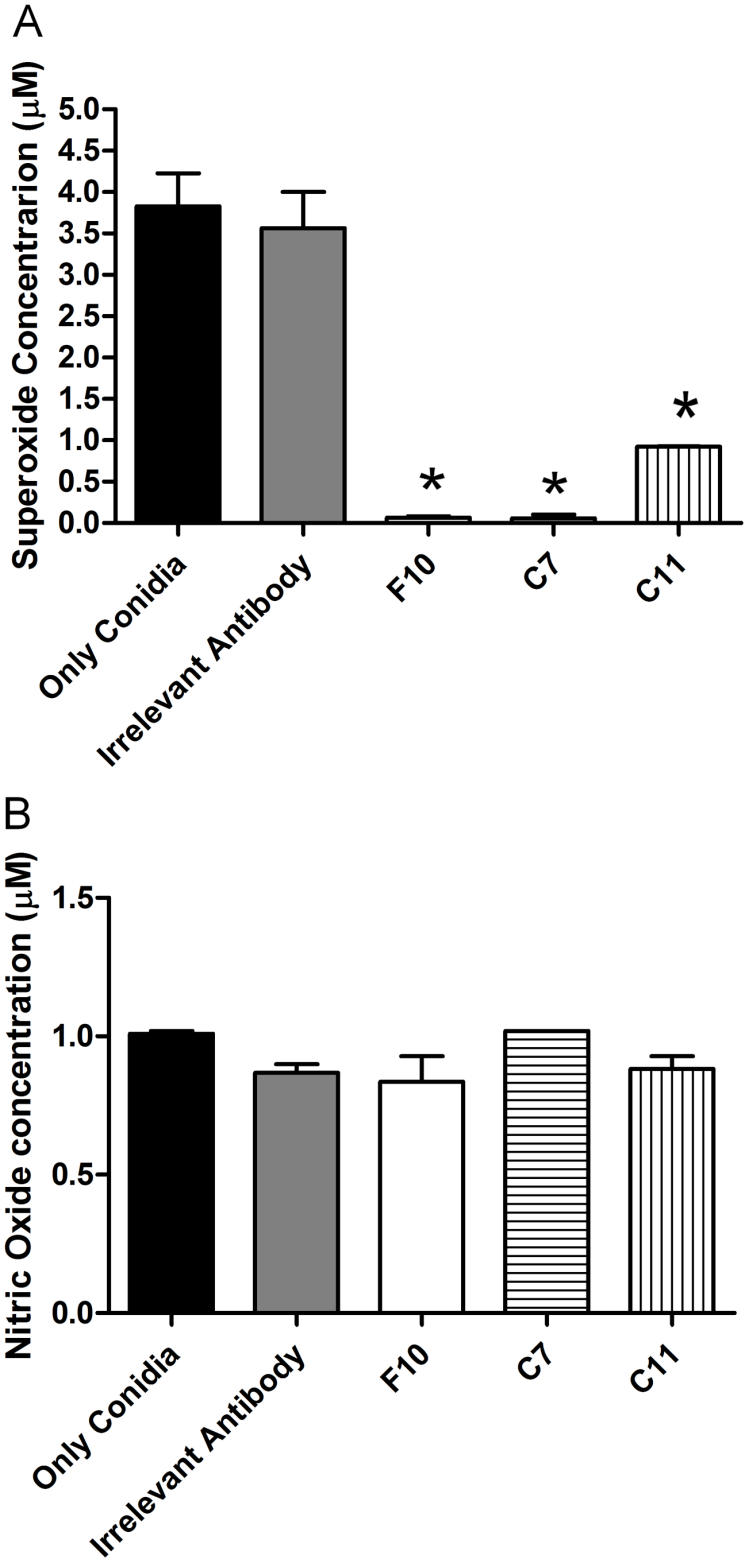
Impact of mAbs to PRM on the release of reactive oxygen species. Opsonization of *S. apiospermum* conidia with mAbs to PRM suppressed the release of superoxide by J774.16 cells (* *P*<0.05) (A). The production of nitric oxide by J774.16 cells was not affected by the presence or absence of mAb (B). The experiments were performed three times.

Addition of mAbs F10, C7 and C11 altered superoxide production by macrophages. A decrease in superoxide production occurred in the presence of the mAbs in comparison with the controls (*p*<0.05) (Figure 6A). In contrast, the release of nitric oxide by J774.16 cells co-cultured with *S. apiospermum* conidia was not affected by opsonization (Figure 6B).

### Survival studies using mAbs to PRM

**Figure 7:**
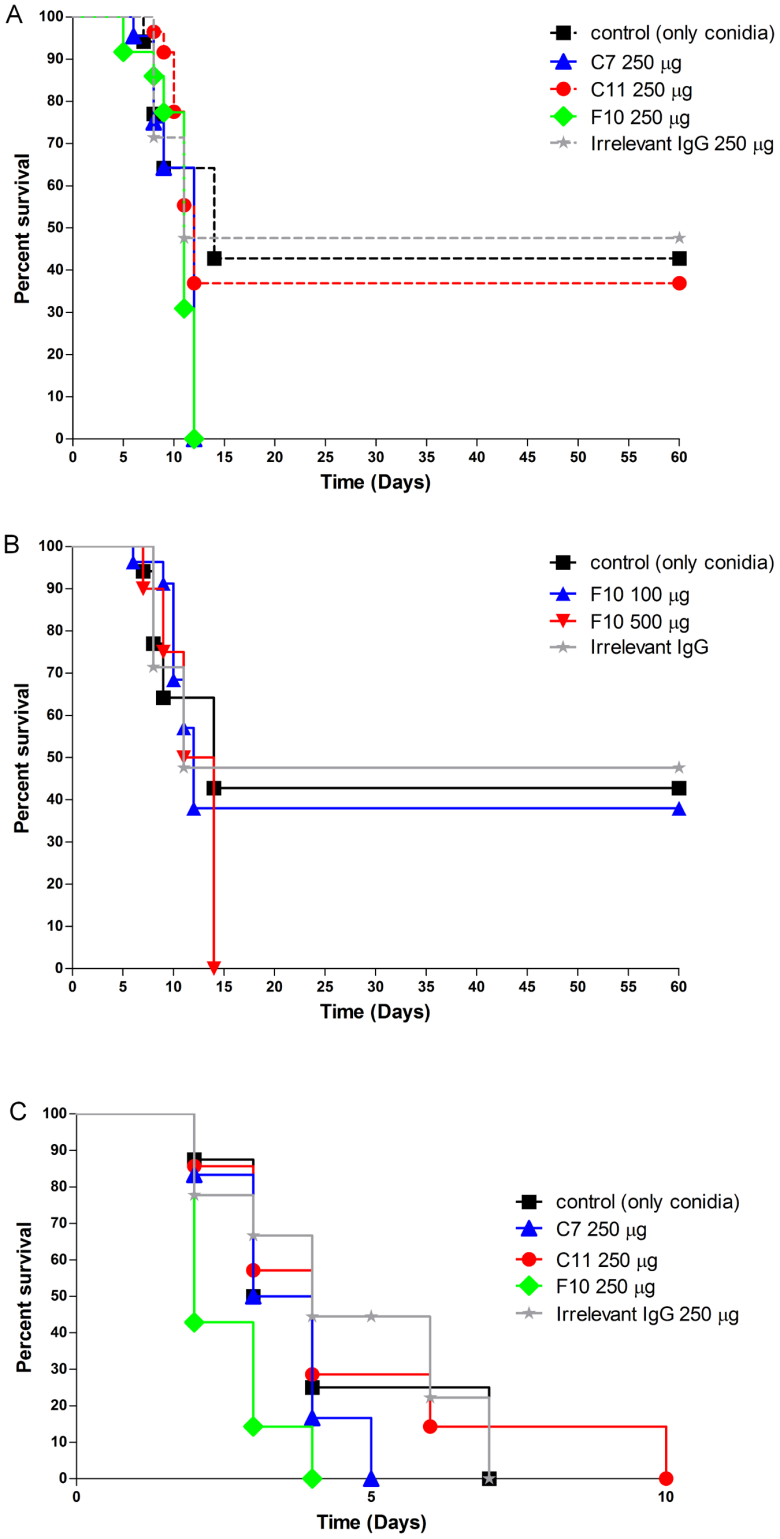
MAbs to PRM affect the pathogenesis of scedosporiosis. Intraperitoneal injection of 250 µg of mAbs F10, C7 and C11 2 h prior to infection significantly decreased survival in intravenous model of infection with *S. apiospermum* conidia (A). Similar results were observed with 100 and 500 µg mAb F10 (B). Intratracheal infection with *S. apiospermum* conidia showed 100% of mortality in all the conditions, but in the presence of 250 µg mAb F10, mice died before the controls groups (C) (*P<0.05). The survival experiments were performed twice.

To determine the effect of the mAbs to PRM in scedosporiosis, mice were treated with mAb to PRM, irrelevant antibody or PBS and then intravenously or intratracheally infected with *S. apiospermum* conidia. In the intravenous model, administration of 250 µg of mAbs F10 or C7 accelerated disease, resulting in 100% mortality by day 12 (p<0.05) (Figure 7A). Although 250 µg of mAbs C11 also enhanced mortality, the difference from controls was not statistically significant. To assess for the possibility of a prozone effect with the dose selected, mice also were treated with 100 or 500 µg of the mAb F10. Administration of 500 µg of mAb F10 resulted in 100% mortality, but 100 µg was not significantly different from controls (Figure 7B).

Intratracheal infection resulted in 100% mortality for each condition examined. MAbs F10 and C7 were able to enhance mortality, but the difference from controls was only significant for mAb F10 (p<0.05) (Figure 7C).

#### Effects of mAbs to PRM on *C. albicans*

*C. albicans* growth was assessed by cultivation of yeasts in YPD broth at 30°C with and without mAbs to PRM. The growth of *C. albicans* increased significantly at 12, 24 and 36 h, respectively, in the presence of mAb F10 compared to controls (p<0.05) (Figure S2A). However, mAbs C7 and C11 had no effect on growth. Additionally, opsonization with the mAbs reduced the phagocytic index by 15%, 40%, and 50% for F10, C7 and C11, respectively, compared to controls (Figure S2B). To further study mAb effect, we examined kidney fungal burdens at 7 days after infection using mAb F10 compared to controls as a measure of dissemination in intravenously infected mice. Kidney CFUs were twice as great in mice pre-treated with mAb F10 compared to mice receiving irrelevant antibody or PBS (p<0.05) (Figure S2C).

## Discussion

New medical technologies and therapies have dramatically amplified the numbers of severely immunosuppressed patients, increasing the risk for disease from several opportunistic yeasts and filamentous fungi [23]. In this context, *S. apiospermum* is emerging as an important human pathogen [23]. The increase in scedosporiasis is especially clinically concerning as numerous *in vitro* studies have shown that antifungal drugs, such as amphotericin B, nystatin, liposomal nystatin, itraconazole, flucytosine, fluconazole, terbinafine and ketoconazole, have low *in vitro* activity against fungi of the *Scedosporium-Pseudallescheria* complex [5], [24]–[25]. The implementation of antibody-based therapeutics remains largely empirical in part because the factors involved in efficacy are often poorly understood and activation of inflammatory pathways by antibodies may be detrimental [26]–[29]. Although we set out to identify mAbs that would protect against *S. apiospermum* disease, our panel of mAb to *S. apiospermum* PRM modified the biology of the fungus to enhance virulence.

The mAbs to PRM bound antigen located on the surface of *S. apiospermum* strain HLPB mycelia and/or conidia. The mAbs also reacted with PRM-like compounds on the cell surface of *C. albicans*, *C. parapsilosis*, *H. capsulatum*, *S. apiospermum* clade 5 and *S. prolificans*. *S. apiospermum* clade 5 and *S. prolificans* both display surface PRM with only minor differences from *S. apiospermum* PRM structures [30]. Structural studies on *S. apiospermum* PRM have shown that α (1→2)-linked mannosyl units are also located in side chains that have α-mannosyl or α-rhamnosyl terminal non-reducing units [31], probably located in the *N*-linked glycan component of the PRM [32]. In the present study, we demonstrated that the carbohydrate portion of the PRM molecule is essential for recognition of the IgG1 mAbs F10, C7 and C11, since PNGase F-treatment of PRM (lacking *N*-glycans) significantly reduced mAb binding. The cross-reactivity with the yeasts is presumably related with the presence of α-1,2-mannosyl residues on the surface of these fungi. By competition ELISA, the mAbs against PRM competed for PRM binding, but they had distinct labeling patters by immunofluorescence. These results suggested that mAbs F10, C7 and C11 recognized very close or overlapping epitopes.

Changes in the cell surface characteristics of resting conidia during swelling and germination may interfere with mAb recognition. In *S. apiospermum*, glucosylceramides are detectable on the surface of mycelia and pseudohyphae but not conidial forms, suggesting a differential expression of these glycoconjugates according to the morphological phase of the fungus [33]. Antibodies against glucosylceramides can modify the transition conidia-mycelium in *S. apiospermum* and the cellular differentiation in *C. albicans* [33]. *Fonsecaea pedrosoi* differentially expresses sialylglycoconjugates and sialidase in distinct morphological stages, producing these molecules in conidial and mycelial forms, but not in sclerotic cells, suggesting that the sialic acid expression in *F. pedrosoi* varies according to the morphological condition [34]. Many virulent strains of *H. capsulatum* possess α-(1,3)-glucan in the yeast cell wall, although it is absent in the mycelial form [35]. This polysaccharide is likewise present in the yeast phase of two other pathogenic dimorphic fungi, including *Paracoccidiodes brasiliensis* [36] and *Blastomyces dermatitidis* [37]. In each of these species, spontaneous variants that have lost their α-(1,3)-glucan have also lost virulence. So, the presence and the native structure of PRM could be different according with the stage of *S. apiospermum* (resting conidia, swollen conidia, hyphae), which could affect mAb binding.

Antibodies can facilitate clearance of fungi from the lungs, bloodstream, or other tissues through a combination of opsonization via the Fc fragment of the antibody and opsonization via classical pathway deposition of C3 [38]. In the present work we decided to investigate the involvement of the mAbs and of the PRM molecule in the phagocytosis of *S. apiospermum* conidia by J774.19 macrophage-like cells. Our results demonstrated the mAbs against PRM reduce the uptake of *S. apiospermum* conidia and *C. albicans* yeasts by J774.16 cells and opsonized conidia have increased intracellular survival. Previous work from our group using HEp2 cells showed that when the conidial cells of *S. apiospermum* were pre-incubated with polyclonal antibodies to PRM, the adherence and endocytosis processes were both inhibited in a dose-dependent manner [10]. These results suggested an active participation of the fungus in the interaction process, since HEp2 cells are considered nonprofessional phagocytic cells. In professional phagocytic cells like macrophages, Fc receptors are constitutively active for phagocytosis [39]. Consequently, it is expected that opsonization of yeasts by mAbs facilitates the ingestion of conidia by macrophages. For example, *H. capsulatum* Hsp60-specific mAbs augment yeast cell phagocytosis by J774.16 macrophage-like cells [17]. However, protective mAbs against the glucuronoxylomannan (GXM) component of the *Cryptococcus neoformans* capsular polysaccharide are not always effective mediators of cryptococcal phagocytosis [40]. The mechanism for differential levels of phagocytosis following opsonization by intact IgG is not known. It is possible that, depending on epitope specificities, alterations of the antigen structure may occur on binding that interfere with the interaction between the bound IgG and macrophage Fc receptors.

Our *in vitro* experiments were done in the absence of sera, which does not allow us to directly address the role of complement in the opsonization via classical pathway deposition of C3. However, the mAbs used in this study were all IgG1 subclass, which does not activate complement well [41]. Other subclasses, especially IgG2a and IgG2b, could contribute to complement-dependent-opsonopahgocytosis of conidia in a milieu with serum. The ability of complement activation by antibodies may be related to their protective effect in infections, as documented in response to encapsulated bacteria [42]–[44]. Interestingly, only 1 of a pair of IgM mAbs to cryptococcal polysaccharide that activated complement *in vitro* were protective in murine models of cryptococcosis [45]. Furthermore, additional mAbs to cryptococcal polysaccharide suppress rather than enhance binding of C3 to the cryptococcal capsule and this suppressive activity is dependent on the antibody isotype and epitope specificity [46]. In addition to isotype, the epitope specificity influences the biological activity of antibodies [47]. Hence, the lack of protection in our model by the IgG1 mAbs to PRM may be due their weak ability to activate classical pathway deposition of C3, but it may be also be associated with blocking C3 binding.

Although the F10 mAb increased intracellular survival, it also enhanced phagolysosomal fusion, which is often associated with increased killing of microbes within these structures [48]. *S. apiospermum* conidia, in the presence or absence of mAbs anti-PRM, were able to destroy macrophages. Interestingly, we found that *S. apiospermum* conidia could readily survive acidic conditions with enhancement of growth. A possible explanation for the observed growth benefit is that *S. apiospermum* proteolytic enzymes (metallopeptidases or metal dependent peptidases) require an acidic pH for optimal activity and these peptidases are key enzymes implicated in microbial metabolism and virulence [1], [49]–[51]. Surprisingly, mAbs C7 and C11 did not significantly enhance phagosomal fusion with lysosomes, which could be related with their different binding properties.

The effect of a mAb depends on several factors, including the targeted antigen, its function, the cell surface density and characteristics of the mAb, including specificity, avidity, and isotype. When a fungal spore germinates, it produces a hypha, which in turn grows by increasing in length through the accumulation of newly formed substances on the hyphal wall [52]. Prevention of some fungal infections presumably requires control of either spore germination and/or hyphal growth. We found that mAbs F10, C7 and C11 enhanced conidial germination compared to controls, indicating that these mAbs may have accelerated the modification of the inner wall structure. The increased metabolic activity shown by MTT analysis of *S. apiospermum* conidia and *C. albicans* yeasts exposed to the mAbs is consistent with the enhancement of cellular processes required for morphogenesis.

Reactive oxygen and nitrogen species can impact fungal growth. Activated bronchoalveolar macrophages and neutrophils can kill *H. capsulatum* by mechanisms dependent on hydrogen peroxide and products of the nitric oxide synthase pathway, whereas fungistasis depends largely on products of the nitric oxide synthase pathway [53]. In cryptococcosis, superoxide dismutase is important because this enzyme can interfere with *C. neoformans* virulence by affecting the fungus growth inside the macrophages [54]. In this context, we decide to evaluate the profile of superoxide and nitric oxide in *S. apiospermum* infection in macrophages cells, when the conidia were opsonized by mAbs F10, C7 and C11. We found that macrophage superoxide production decreased in the presence of conidia treated with mAbs compared with controls. Although nitric oxide is another important part of the oxidative attack directed against many microbes, the production of nitric oxide was not affected by the mAbs to PRM, in comparison with controls [55]–[56].

MAbs have previously been described to enhance experimental systemic fungal disease, such as mAb 7B6 for *H. capsulatum* [17] and mAb 13F1 for *C. neoformans* [45], [57]. We observed that mAbs F10 and C7, but not C11, could enhance the disease in murine models of intravenous and intratracheal infection. Similarly, mice pre-treated with mAb F10 and infected with *C. albicans* yeasts had higher fungal burdens in the kidneys at 7 days after infection compared to control mice. Additionally, the mAbs to PRM could enhance *C. albicans* growth and decrease the phagocytosis of *C. albicans* yeasts by J774.16 macrophages relative to controls.

In the present study, we demonstrated that mAbs to PRM are either non-protective or disease-enhancing in our *S. apiospermum* infection models. Hence, administration of mAbs that bind PRM on the surface of *S. apiospermum* conidia decreases phagocytosis, increases intracellular survival, and increases germination that results in a survival advantage for the fungus during host-pathogen interactions. We additionally found that the mAbs interacted with diverse fungal species and modified the virulence of *C. albicans*. Hence, antibodies to glycocongugates may significantly impact the pathobiology of many fungi. Further studies are required to gain a more detailed understanding of the antagonist behavior of the mAbs to PRM in *S. apiospermum* disease and explore the impact of antibody responses to these important cellular structures.

## Supporting Information

Figure S1 Representative curves showing mAbs binding with different fungi. *S. apiospermum* clade 5 (A) and *S. prolificans* (B) conidia, and *H. capsulatum* (C), *C. albicans* (D), and *C. parapsilosis* (E) yeasts. The ELISA assays were done in triplicate, three times.
(0.81 MB TIF)

Figure S2 MAbs can affect *C. albicans* growth, phagocytosis and murine infection. Growth of *C. albicans* increases in the presence of mAb F10 compared to controls but not in the presence of mAbs C7 and C11 (A). Phagocytosis of *C. albicans* was reduced in the presence of yeasts opsonized with mAbs to PRM (B). The experiments were performed three times (* P<0.05). Numbers of CFU in kidneys at 7 days after sublethal intranasal challenge with 1×10^6^ *C. albicans* yeast cells for mice treated intraperitoneally with mAb F10, irrelevant mAb, or PBS (C). *P<0.05.
(0.62 MB TIF)

Video S1 *S. apiospermum* conidia are able to destroy J774.16 during the infection. In the movie, it is possible to observe that *S. apiospermum* conidia can destroy macrophages during the course of infection. Images were collected at 10×.
(2.54 MB AVI)
