# Supplementary figures and images for: Monoclonal Antibodies Against Peptidorhamnomannans of Scedosporium apiospermum Enhance the Pathogenicity of the Fungus
Source: PLoS Negl Trop Dis. 2010 Oct 19;4(10):e853. doi: 10.1371/journal.pntd.0000853 (PMC2957425; doi:10.1371/journal.pntd.0000853)

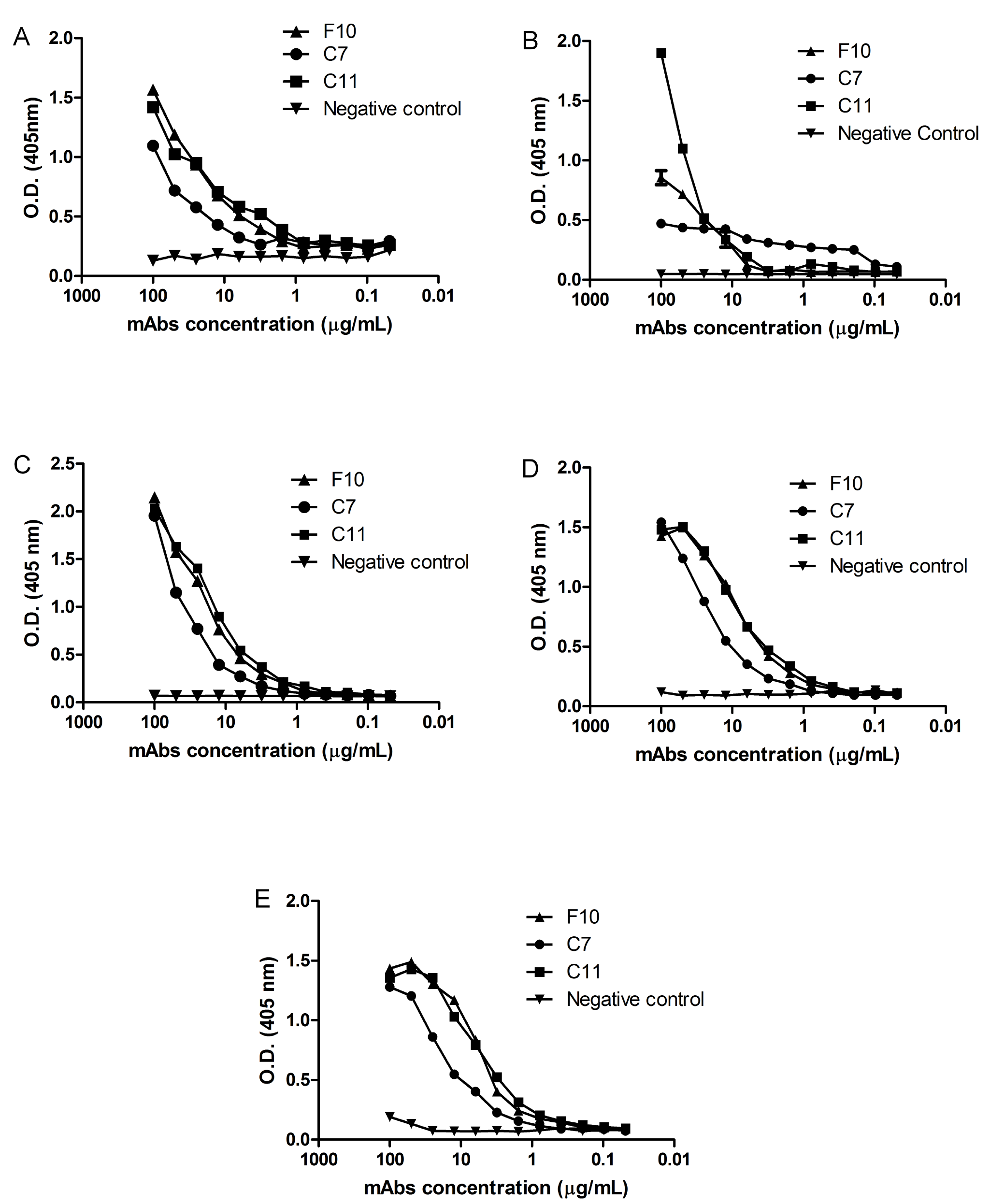

Supplement: Figure S1 — Representative curves showing mAbs binding with different fungi. S. apiospermum clade 5 (A) and S. prolificans (B) conidia, and H. capsulatum (C), C. albicans (D), and C. parapsilosis (E) yeasts. The ELISA assays were done in triplicate, three times. (0.81 MB TIF) [file pntd.0000853.s001.tif]

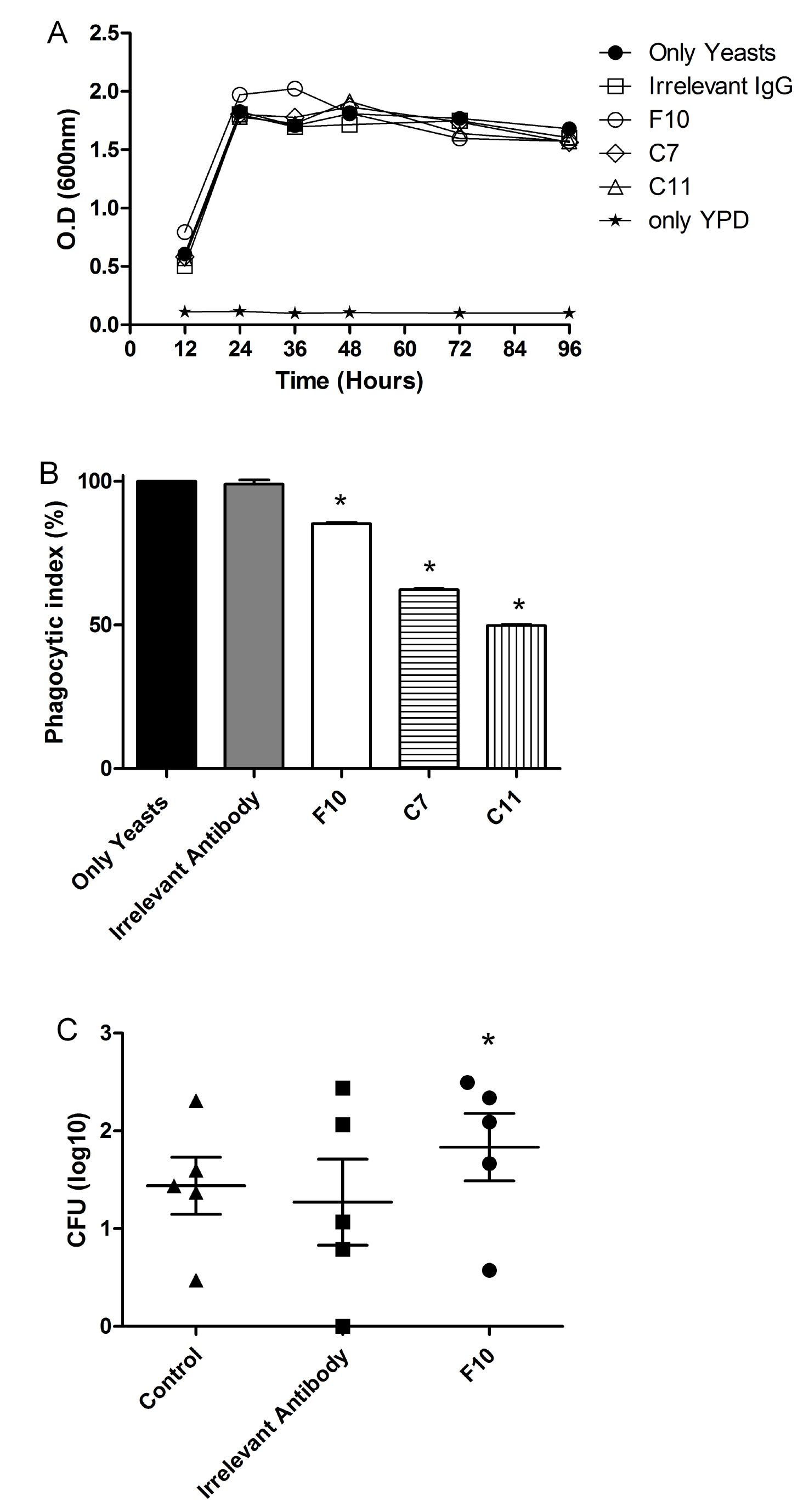

Supplement: Figure S2 — MAbs can affect C. albicans growth, phagocytosis and murine infection. Growth of C. albicans increases in the presence of mAb F10 compared to controls but not in the presence of mAbs C7 and C11 (A). Phagocytosis of C. albicans was reduced in the presence of yeasts opsonized with mAbs to PRM (B). The experiments were performed three times (* P<0.05). Numbers of CFU in kidneys at 7 days after sublethal intranasal challenge with 1×10^6^ C. albicans yeast cells for mice treated intraperitoneally with mAb F10, irrelevant mAb, or PBS (C). *P<0.05. (0.62 MB TIF) [file pntd.0000853.s002.tif]
